# Reflections on an International Research Immersion Field Study as a High Impact Practice to Produce Publishable Papers by Underrepresented Undergraduates

**DOI:** 10.3389/fpsyg.2019.00601

**Published:** 2019-03-27

**Authors:** Heather M. Hill, Melissa Karlin

**Affiliations:** ^1^Department of Psychology, St. Mary’s University, San Antonio, TX, United States; ^2^Environmental Science, St. Mary’s University, San Antonio, TX, United States

**Keywords:** high impact practices, immersion experience, field study, study abroad, undergraduate research, psychology

## Abstract

Engaging undergraduates in publishable research is challenging. Skills including researching topics, statistical knowledge, and writing abilities are necessary; however, students often face time constraints or financial challenges that impede them from engaging in these experiences. Conducting research with underrepresented students can be an even bigger challenge, as these groups are known to face additional financial or family burdens that the traditional student does not face. This essay reports on the development of an international field study with the goal of producing publishable research by undergraduates. To date, 27 students (68% Hispanic, 52% first generation) have participated in a week-long immersion field experience in Roatán, Honduras. As an interdisciplinary field study, students were exposed to animal behavior, ecology concepts, and research methods through a two-course sequence that incorporated the field experience. In this essay, we share our best practices for conducting a field study with students from underrepresented populations with the goal of producing publishable research. We include the evolution of the course curriculum that was informed by self-reported student experiences and a brief description of some of the projects students designed. Students reported that the field experience highlighted the importance of adjusting research plans and expectations. Ultimately, this program exposed students to advantages and disadvantages of conducting field research while increasing confidence in their ability to conduct effective and meaningful research. A minimum of two semesters may be needed to create publishable research projects and 1 week of data collection is not sufficient for successful research projects.

## Introduction

Engaging undergraduates in research at a minority-serving institution has many challenges. Competing factors such as family expectations, financial constraints, lack of awareness regarding the importance of research, and the underdevelopment of critical research skills are some of the obstacles encountered by faculty working with underrepresented students (Ortiz, 2004; Bridges et al., 2008; Kuh, 2008; Bangera and Brownell, 2014). These obstacles may be amplified if the institution is also a primarily undergraduate teaching university, where research is expected of faculty in addition to teaching and service duties, but the financial support for research may be lacking (Smith and Brown, 2012; Anastasio, 2016). However, high impact practices such as field studies and immersion experiences are known to be both transformative and productive for participants and need to be encouraged for all students, regardless of circumstances (Barnett, 1997; Lopatto, 2007; Jones et al., 2010; Kuh et al., 2010; Finley and McNair, 2013; Bangera and Brownell, 2014). Our research immersion program combines curriculum-based research with an international, field immersion experience. The purpose of this essay is to share our best practices for conducting an interdisciplinary, week-long, international field immersion study supported by curriculum-based research experiences with the goal of collecting data for a publishable research project.

## Logistics of the Field Course

**Figure 1:**
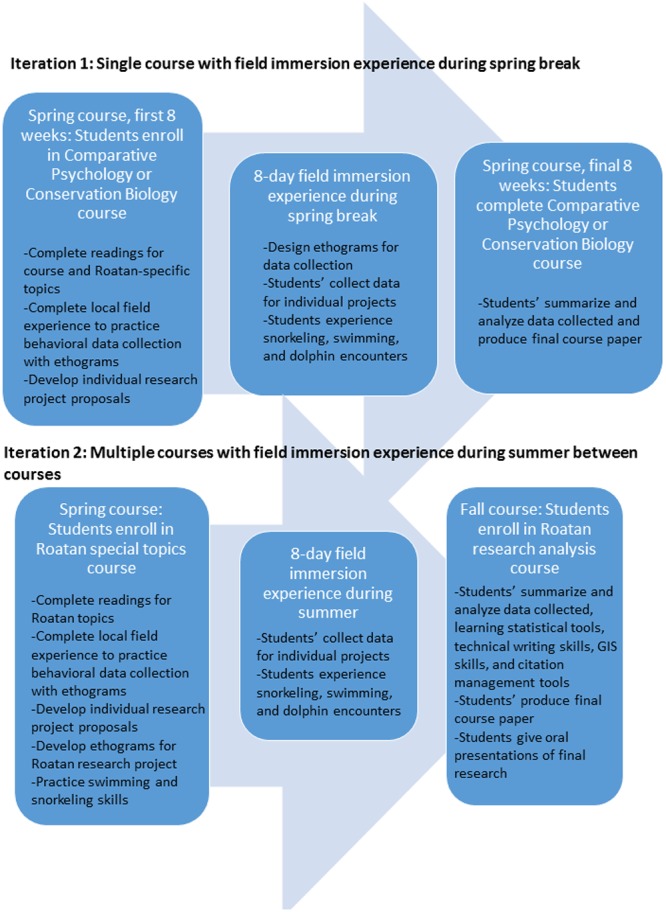
Flow process of Iteration 1 and 2. Neither iteration resulted in publishable research, mainly due to quantity and quality of data collected. However, Iteration 2 enabled the students more time to conduct the necessary background research, learn essential research skills, and practice presenting their research in an oral format. Based on feedback from the students’ in each iteration, and the progress made in research skills and results in Iteration 2, we recommend future experiences incorporate longer field immersion experiences.

To date, we have completed two field studies on the island of Roatán, Honduras with a total of 27 undergraduate students, including two repeat students. Of the 25 unique students, 68% self-identified as Hispanic, and 52% as first generation/low income. Our institution, St. Mary’s University located in south central Texas, serves over 2,000 undergraduates and is a Hispanic Serving Institution (HSI) with a high percentage of first generation and low-income students. Our recruitment efforts reflect the distribution of our university and the disciplines of the two authors. The field study is interdisciplinary with emphases on comparative psychology and environmental science, and students had the opportunity to design independent research projects involving animal behavior and cognition with bottlenose dolphins as the study animal, or conservation biology/environmental science topics related to the marine environment. The majority of the students had taken either statistics and/or research methods courses or have been involved in smaller, independent research projects prior to attending the field study. Completing at least one of these courses or a previous research experience is necessary for students to create a research study that is publishable. The field study has gone through two iterations thus far with important lessons learned from the inaugural field study that were then implemented in the second iteration to try and increase the likelihood of successfully completing a publishable research project (Figure 1 illustrates the two iterations).

### Iteration 1

In the first iteration, we had 13 students, of which 9 identified as Hispanic and 1 additional identified as non-Caucasian; all 13 were female. The students took either conservation biology or comparative psychology in the spring semester, which was paired with a laboratory course that consisted of the field study during spring break. Both courses were coordinated prior to spring break so that students in each course attending the field study could learn the skills necessary to complete their research projects while in Roatán. The students who were not attending the spring break field study completed similar research projects but with local resources (e.g., local zoo, campus, or surrounding natural parks and reserves). Students in both classes read original, peer-reviewed scientific literature on topics involving animal behavior that targeted constructs that could be examined in the field, conservation issues and efforts (with special emphasis on the tropics), ecological diversity, and research methods for behavioral and environmental sciences. With guidance from the coordinator of the field study and the two authors, the students also developed research proposals for projects to be collected during the 6-day field immersion period. Students developed their projects independently of one another, although topics and data collection methodology could overlap. Example project topics included: Species diversity of seagrass in Anthony’s Key and Bailey’s Key in Sandy Bay, Roatán, Honduras; and type and frequency of play behavior by dolphins and frequency of pair swims in same-sex and mixed-sex dolphin dyads. Once in Roatán, all students assisted on data collection for all projects so that they could be cross-trained on the different methods employed by each discipline; however, each student was using the data collected for their own, unique research project. In addition to data collection for their projects, the field study experience included lectures, discussions, and special presentations by local educators and trainers on topics unique to the island and marine ecosystem, snorkeling, and kayaking every day. Following the field study, the participants and their classmates completed their data analyses and formal write-ups as the final project requirement of the two courses. Ideally, any of these projects could have been publishable if enough data had been gathered. Unfortunately, given the individual nature of the projects and the time available to collect data during the field study itself, the majority of the projects did not have sufficient data to warrant a publication. However, several of the projects were presented at our university’s symposium that spring and one conservation biology project investigating soil and water quality on Roatán was presented at an international conference, earning an outstanding undergraduate research award and travel grant.

### Iteration 2

Based on the semester-long style for iteration 1, which is a common approach for many field studies, and the feedback provided by the 13 students, we modified the format and required a two-semester commitment by the students participating in the field study. We had 14 students in the second iteration, of which 9 identified as Hispanic and an additional 1 identified as not Caucasian, and we had 10 females. The first semester, the field study students enrolled in a special topics course for 3 h of credit in the spring. The format of this course was the same as in the first iteration, meaning that all required reading was completed before attending the field study (which had been a challenge in the first iteration since the field study occurred 8 weeks into the semester), and students were required to develop their individual research projects in collaboration with another classmate or two. At the end of this first course, students had a well-defined plan for data collection and an independent research proposal. All proposals had been guided and were approved by H. Hill and M. Karlin, the instructors of the field study. Example project topics included: Estimating marine specie richness and evenness of seagrass habitat in Roatán, Honduras; and activity level of dolphins before, during, and after the presence of a swimmer or number of dolphins in a designated safe zone area during a swim program. The field study itself occurred later in the summer the week before the fall semester began. Upon the students’ return from the field study, students began a second special topics course, which emphasized advanced research methods and ended with a drafted research manuscript and oral presentation. Students were also required to present their field study research in either oral or poster format at the university’s research symposium in the spring following the field study experience.

## Summary of Student Experiences

All students in both field studies completed a post-field study survey immediately at the end of the week. The second field study students also completed a pre-field study survey at the beginning of the spring semester course. The surveys included questions specific to the program we developed as well as four previously validated instruments: Interest in Research Questionnaire (Bishop and Bieschke, 1998); Research Self-Efficacy Scale (Greeley et al., 1989); general self-efficacy (NGSE, Chen et al., 2001); New Ecological Paradigm scale (NEP, Dunlap et al., 2000). The student free responses supported previous research findings for international immersion experiences (Barnett, 1997; Lopatto, 2007; Jones et al., 2010; Kuh et al., 2010; Finley and McNair, 2013; Bangera and Brownell, 2014): our students reported testing and pushing their personal boundaries and comfort zones while increasing their confidence and becoming more aware of the culture, economy, and pressures of those in the country they visited (Tables 1–4). The results from the quantitative data from these surveys are available for review (Karlin and Hill, unpublished). Many of the students reported that the field experience highlighted the importance of adjusting research plans and expectations (Tables 1–4). Whether the students developed their ethograms for data collection during the first 2 days of the field study (first iteration) or prior to the field study (second iteration), all the students had to modify their selected research projects to account for field conditions. Having never conducted their own research study or developed a study with live subjects in an ever-changing field conditions (e.g., weather changes, availability of animals for data collection, access to locations for data), all the students reported being overwhelmed initially (Tables 2–4). During and following both field studies, the students reported that working in a small group setting with peers that were conducting similar research helped them to develop, refine, and implement their projects more successfully. Example responses from the post-survey responses include (Tables 1–4): “This study offered experiences that one may not normally have the time or money to do after he/she has graduated from school.”; “This field study was absolutely transformative, I was able to gain critical research experience while also making lots of new friends and priceless memories.”; “… I feel like my learning capabilities have transformed into something much bigger than a classroom.”

**Table 1:** Summary of themes and comments produced by field study students regarding goal achievement.

	Number of responses		
Cohort	per theme categories^a^		Comment
	Achieved goals	Did not achieve goals	
2016 (*n* = 13)	12	1	Everything I did was something I never thought I would do.
			I had a great time and was able to learn more about the island and the country through some of the articles about Roatán and their conservation efforts.
			I feel like the trip enhanced my education and gave me experiences I wouldn’t get anywhere else. It’s also a unique experience for an undergraduate and I feel lucky that I got it.
			However, I did accomplish and a lot and learned even more.
			I feel like I did it was a wonderful experience!
			So, it was pretty much AMAZING!
			I conquered many fears coming on this trip and I have grown so much from this experience. I am forever grateful to the wonderful people that made it possible for me to even be here. I feel accomplished and ready to take on the world.
			It was purely amazing I overcame many many fears and did thing that I thought I would never have the opportunity or bravery to do this was great thank you both for making this possible.
2018 (*n* = 14)	13	1	I wanted to identify weak points and needed improvements in the brainstorm process for designing a research study. From this, I wished to learn limitations, foster a more rational and developed thinking process for scientific understanding, and learn how to step back and take different ideas and challenges into new modifications and procedures. It felt quite different, as I wanted to know how to identify measures that may have been missed in the research process, such as stimulus effects or factors that may have been overlooked in the data collection process. It felt good to be challenged, and to understand why it was challenging.
			but i am sure i accomplished them.
			This trip showed me a lot, it taught me a lot and it gave me amazing memories that will last a lifetime.
			Yes, it was very fulfilling to be able to accomplish so much in such a short amount of time.
			Yes! It felt good to be able to come ok out of this experience not only well rested and relaxed but so much smarter and experienced.
			I feel quite accomplished. I gathered sufficient data, made connections with the group got a refresher about limitations and live data gathering.
			but I like to think that I got everything I possibly could out of this trip.
			I possibly could out of this trip. I learned so much about research and marine life.
			collect enough data to publish a paper. We did not accomplish this, which was sad but we can always right a methodology for others to continue.
			identify all of the dolphins. I was unable to do this, but as the week went on I got a lot better, which was rewarding.
			learn how to collect data on dolphins from the surface level compared to under water. We learned of many different ways to collect data from the surface level from our classmates and we learned how to collect data from the underwater camera.
			Carry out a research plan (able to problem solve when conditions could potentially change) on my own.
			^∗^Experience traveling with a group.
			^∗^Delegate responsibilities to other team members.
			^∗^Experience the marine biodiversity.
			All goals were achieved and it felt enriching and rewarding.
			I believe I did reach the 4 goals I set for this week and that felt quite fulfilling.
			I believe I achieved my goals and it felt amazing. I feel a lot more confident in myself and in my studies because this trip has helped me take the first step to overcoming some of my fears and this included research.

*Question (post-survey only): Cohort 1 – Did you achieve the 4 goals you set for this week? Explain how it felt. Cohort 2 – You identified four goals you wanted to accomplish during the field study. Did you achieve the four goals you set for this week? Explain how it felt. ^*a*^Not all students provided full responses. Student responses were corrected for typographic errors only*.

**Table 2:** Summary of themes and comments produced by field study students regarding preparing for the field study.

	Number of responses			
Cohort	per theme categories^a^			Comment
	Read papers before	Develop projects more fully	Practice dolphin ID	
2016 (*n* = 13)	8	1	2	Read and summarize all the articles in advance so they can get to bed earlier.
				If readings are not done for class, read far ahead.
				Take the time to read the summaries briefly and starts summaries before the trip.
				Read and summarize all your articles before the trip, be flexible and prepared to change your projects.
				It should be definitely a 3-h class. Having all the articles read beforehand will definitely make the Roatán trip more enjoyable.
				read a lot of background on the Island itself also on what you will chose to study.
				Do your homework before you come.
				Read and summarize all the articles in advance so they can get to bed earlier.
2018 (*n* = 14)	4	6	2	Along with reading academic articles on Roatán, look at social blogs and forums to learn more about Roatán to ensure you are prepared.
				Bring copies of articles/notes of articles; paper and writing utensils.
				always have everything for the day prepared the night before.
				Read and brainstorm ideas more carefully. Most importantly, collaborate efficiently and effectively.
				I would say if they are planning to collect data on dolphin behavior it would be good to familiarize themselves with the different kinds of behavior that they display.
				refresh yourself on the articles and research methodology.
				Review video of the dolphins prior to research question.
				going to the lab and practice coding.
				going to the spring class and reading the articles.
				Take the articles with you on the trip so you can reference them at night or during the lectures.
				students make a detailed and specific ethogram so it’s less work once they start collecting data.
				Think of a few factors that may impact your project and think of back up plans for those factors.

*Question (post-survey only): Cohort 1 – What would you recommend to help students prepare for the field study in the future?; Cohort 2 – What would you recommend to help students prepare for the field study in the future? ^*a*^Not all students provided responses that fell into each theme. Also, theme categories were not mutually exclusive, and a student could contribute to multiple categories. The numbers will not add up to the sample totals for these reasons. Student responses were corrected for typographic errors only*.

**Table 3:** Summary of themes and comments produced by field study students cohort 1 regarding recommended changes to field study.

	Number of responses		
Cohort	per theme categories^a^		Comment
	Class should have students read papers before	Class should develop projects more fully	
2016 (*n* = 13)	4	3	There was too much activity happening back to back and adding class time and discussion to it was hard. I think we were too tired and stressed out trying to get school work done that we missed time enjoying the island and the things around us.
			We got a feel of the environmental science folks way of doing things as they also got a feel of what we do.
			By the time we were done with everything, we were physically and mentally tired and had to pull into reserve energy to attempt the articles enough to somewhat comprehend them enough to remember them in the morning.
			There were a lot of changes at the beginning or just before the trip with our research papers. The conservation bio class completely changed their projects and a lot of the comparative psych class had to change their projects (mostly because of the absence of calves or other issues). It would have been good if we’d been able to find out the population before the trip.
			I recommend for this trip to be 2 weeks long to get the ids down and have more time to get data without being stressed.

*Question (post-survey only): Cohort 1 – If there is one thing that could be done differently, what would you recommend? ^*a*^Not all students provided responses that fell into each theme. Also, theme categories were not mutually exclusive, and a student could contribute to multiple categories. The numbers will not add up to the sample totals for these reasons. Student responses were corrected for typographic errors only*.

**Table 4:** Summary of themes and comments produced by field study students cohort 2 regarding the transformative nature of the experience.

	Number of responses			
Cohort	per yes/no question^a^		Number of responses per how/why theme categories^a^				Comment
	Transformative experience	Not a transformative experience	Overcame fears	Connected with nature	Research experience	Connected with others	
2018 (*n* = 14)	12	1	3	5	7	4	YES SO TRANSFORMATIVE! I have so many feelings for dolphins and I want to learn more and more about them :-).
							Yes. This study offered experiences that one may not normally have the time or money to do after he/she has graduated from school. These experiences, I feel, offer students a deeper connection and/or appreciation for nature, and especially marine ecosystems.
							Yes, it is fun, informative, provides research experience, and makes you feel close to the group of people you go with.
							Yes. It gave me steps to understand why a research project could need improvement or replication. I learned many new terms, as well as ideas and insights that will help me in the future. Most importantly, it provided knowledge to take the skills I have even further in such a field of study.
							I would describe this field study as transformative, because I learned a lot of new things that I don’t think I would have without this trip. It was one of the most beautiful places I have ever seen!
							This field study was absolutely transformative, I was able to gain critical research experience while also making lots of new friends and priceless memories.
							Yes I’ve never had a chance to do a field study and to be able to go in and say “I did this” was awesome
							I wouldn’t say transformative but it was a great lesson. It taught us to the down falls and benefits of having real life data collection.
							Yes! I got an experience with marine life that I have never had before. I felt in touch with the outdoors and made some amazing friends.
							Yes, because of the knowledge I now have about dolphins, but even deeper the knowledge I have about myself. This trip takes most people out of their comfort zone and helps them grow as a person. Not only are you learning about the ocean and the animals it entails, but you are also learning about yourself and the future and how you can do research in this field.
							Yes, I’ve extended my self-preservation ideology to other individuals lol.
							Yes, being able to have that experience with the coral reef helped me to really appreciate its role and the role of conservation efforts.
							Yes, because I feel like my learning capabilities have transformed into something much bigger than a classroom.
							Extremely, this trip was all out of my comfort zone but it taught me so much and I feel like my outlook on what I may want to do has changed in a good way. By this, I mean that this trip allowed me to become more outgoing and helped me learn to controls some of my reactions to my fears.

*Question (post-survey only): Cohort 2 – Would you describe this field study transformative? If so, how and why? ^*a*^Not all students provided responses that fell into each theme. Also, theme categories were not mutually exclusive, and a student could contribute to multiple categories. The numbers will not add up to the sample totals for these reasons. Student responses were corrected for typographic errors only*.

## Financial Considerations

During iteration 1, the program fees were incorporated into the laboratory course fee that only the students participating in the immersion experience were enrolled. Therefore, students were able to use their financial aid packages to cover the expenses associated with the experience. Students also held a number of fundraisers prior to the immersion experience, to try and offset some costs. The amount of money raised was minimal; however, it did help to bring the students together and work as a team prior to the trip to Roatán.

During iteration 2, the program fees were incorporated into the spring Roatán class that only the student attending the immersion program were enrolled. Like iteration 1, this meant that the students could use their financial aid packages to cover the costs. However, unlike in iteration 1, the authors wrote and were awarded an internal research grant, the purpose of which was to report on the successes of creating this immersion program. The majority of this award was diverted to the students enrolled in the immersion program to help offset some costs (approximately 20% of the total costs per student).

## Lessons Learned

Ultimately, these two field immersion courses point to the conclusion that to produce a publishable research project, the students need a minimum of two semesters to refine skills previously learned in pre-requisite courses/research projects, develop the research project, analyze the data, and write an initial draft of the paper. Highly motivated students may be able to complete a final publishable product by the end of the second semester with a course devoted to preparing the paper, but most students will likely need a third semester devoted to revising and refining the initial draft to prepare for submission to a journal. Moreover, the data collected during a week-long field study that involves so many other components (in this case, snorkeling trips, discussions, lectures, data processing time) is not sufficient time for meaningful parametric analyses, which then means students must learn about non-parametric statistics while trying to process their data. This limitation may be addressed if students work as teams and collect data across multiple individuals, but this solution has its own issues, namely reliable data collection. It took the students 2 days of practice before they were comfortable with collecting data officially for the project, which limited their data collection opportunities. No publishable research resulted from either iteration (Figure 1). We believe that the students’ can develop successful research ideas; however, the key is the amount of data collected and how much time is spent in the field immersion. Based on our results after 2 iterations, we are recommending at least 2 weeks of data collection, in addition to allowing students to work in groups. These modifications will allow for additional time to collect data, and additional division of data collection amongst the group members, ultimately increasing the sample size of their data. Several students identified working as part as a group one of the elements they considered helpful. The additional time will also partially alleviate issues with uncontrollable field conditions. In our field study, the students were allowed to collect data in teams because their targeted datasets often overlapped, but they had to develop their own hypotheses so that they could write individual papers. It may have been more efficient to allow the students to work as a team on the same project topic as well as for writing the final research paper. Previous research has suggested that working in teams facilitates the research process and makes it less overwhelming (Love et al., 2007).

## Future Directions

Overall, the field immersion experience at an international location has been met with enthusiasm and increased interest by our student population, which is majority minority and underrepresented. In addition to self-reporting increased confidence in their research abilities and enthusiasm for conducting field research (reported in the qualitative comments presented in this essay and the quantitative data Karlin and Hill, unpublished), the international setting and novel experiences led students to report being surprised at the unexpected bonds they developed with their classmates in such a short period (Tables 1, 4). To increase diversity in field study or research experiences, we recommend that instructors recruit from underrepresented student-serving programs such as McNair, MARC-U^∗^STAR, or other TRIO support programs. If instructors give students at least a year to plan for a similar experience, the financial burden can be alleviated by spreading out payments or using financial aid. Some study abroad offices also offer scholarships.

Incorporating a special topics course that was dedicated to the field experience itself was critical in both preparing the students for the field experience (e.g., practice swimming and snorkeling) and developing the research projects more fully. While having the pre-requisite class of either comparative psychology or conservation biology with a laboratory course (iteration 1) was helpful, we found that students felt rushed and more overwhelmed trying to read all of the field study content and prepare a proposal at the same time as managing the rest of the course content in the first 8 weeks of the semester (Figure 1 and Tables 1–3). This iteration was not conducive to creating publishable data (i.e., insufficient data collection period, minimal manuscript preparation time). By requiring a two-course sequence with recommended pre-requisite classes/experiences (e.g., statistics, research methods, comparative psychology, conservation biology, relevant research experience), the second iteration was more successful in creating projects that were potentially publishable. Even with the changes employed in iteration 2, we still had issues with students having sufficient data for a publishable manuscript.

Becoming engaged in a research project, having the time to devote to sufficient data collection, and then writing a publishable paper on an original research project is extremely challenging for upper-division undergraduates in general, and especially at schools with characteristics such as ours. Having the second full semester allows the students to develop their projects more formally for the potential to publish in an external research journal, if they collected sufficient data during the field study. In response to these two experiences, our next iteration will involve a three-course sequence with the field study and data collection experience occurring immediately after the spring semester rather than immediately before the fall semester. The first course will be either comparative psychology or conservation biology, which will be the pre-requisite courses to enroll in the field study special topics course the following semester. These are courses that also count toward their major degree requirements and do not add to their degree load. These pre-requisites will allow the students to learn and practice methods they may implement in their research projects while also learning about different research topics. The second course will involve preparing a research proposal and data collection methodology. Students will be allowed to work in groups and prepare a group research proposal, so that during the field immersion experience they have the opportunity to collect more data than was possible during our previous two iterations. Within this semester-long -field-study course, students will also acquire more comfort with swimming, snorkeling, and data collection techniques to be employed in the field, which was a highly recommended component of our second iteration. Following the second course and the field study experience, the third course will mirror the second course format in our second iteration, and all the students will analyze their data and prepare manuscripts for submission to an appropriate journal and/or conference. All students within each research group would serve as authors (arranged alphabetically by last name) on any papers or presentations that result from the research. Although it may take a little time to set up, we believe this model would be the most efficient process to produce publishable undergraduate research.

One other model that we are currently considering that may facilitate undergraduate research projects into publishable formats is to have student groups use data on three or four previously established projects with reliable and validated protocols, but develop and test their own novel hypotheses while adding to the existing data archive. Whichever path one selects, an immersion field study, such as the one we have conducted, exposes students from all backgrounds to the advantages and disadvantages of conducting field research while increasing their confidence and beliefs in their ability to conduct effective and meaningful research that is ultimately publishable. Adequate preparation through a research-based curriculum and sufficient time collecting data and refining methodologies are key to producing publishable research for undergraduates.

## Ethics Statement

Students gave permission to share their anonymous responses and approval to conduct the study was granted by St. Mary’s University Institutional Review Board.

## Author Contributions

HH initially drafted the manuscript, co-taught the field study, one class, and collected and analyzed the data for the original study cited in this study. MK edited the manuscript, co-taught the field study, one class, and analyzed the data for the original study cited in this study.

## Conflict of Interest Statement

The authors declare that the research was conducted in the absence of any commercial or financial relationships that could be construed as a potential conflict of interest.
